# Fructose and methylglyoxal-induced glycation alters structural and functional properties of salivary proteins, albumin and lysozyme

**DOI:** 10.1371/journal.pone.0262369

**Published:** 2022-01-21

**Authors:** Mariane Yumiko Muraoka, Allisson Benatti Justino, Douglas Carvalho Caixeta, Julia Silveira Queiroz, Robinson Sabino-Silva, Foued Salmen Espindola

**Affiliations:** 1 Biochemistry and Molecular Biology Laboratory, Institute of Biotechnology, Federal University of Uberlandia, Uberlandia, Minas Gerais, Brazil; 2 Innovation Center in Salivary Diagnostic and Nanotheranostics, Institute of Biomedical Sciences, Federal University of Uberlandia, Uberlandia, Minas Gerais, Brazil; University of Colorado Denver School of Medicine, UNITED STATES

## Abstract

Glycation process refers to reactions between reduction sugars and amino acids that can lead to formation of advanced glycation end products (AGEs) which are related to changes in chemical and functional properties of biological structures that accumulate during aging and diseases. The aim of this study was to perform and analyze in vitro glycation by fructose and methylglyoxal (MGO) using salivary fluid, albumin, lysozyme, and salivary α-amylase (sAA). Glycation effect was analyzed by biochemical and spectroscopic methods. The results were obtained by fluorescence analysis, infrared spectroscopy (total attenuated reflection—Fourier transform, ATR-FTIR) followed by multivariate analysis of principal components (PCA), protein profile, immunodetection, enzymatic activity and oxidative damage to proteins. Fluorescence increased in all glycated samples, except in saliva with fructose. The ATR-FTIR spectra and PCA analysis showed structural changes related to the vibrational mode of glycation of albumin, lysozyme, and salivary proteins. Glycation increased the relative molecular mass (Mr) in protein profile of albumin and lysozyme. Saliva showed a decrease in band intensity when glycated. The analysis of sAA immunoblotting indicated a relative reduction in intensity of its correspondent Mr after sAA glycation; and a decrease in its enzymatic activity was observed. Carbonylation levels increased in all glycated samples, except for saliva with fructose. Thiol content decreased only for glycated lysozyme and saliva with MGO. Therefore, glycation of salivary fluid and sAA may have the potential to identify products derived by glycation process. This opens perspectives for further studies on the use of saliva, an easy and non-invasive collection fluid, to monitor glycated proteins in the aging process and evolution of diseases.

## Introduction

Glycation process refers to a sequence of non-enzymatic reactions that begin when reducing sugars such as glucose and fructose react with nucleophilic groups of amino acids from proteins, lipids or nucleic acids forming Schiff bases and Amadori products to produce advanced glycation end products (AGEs) [1,2]. In addition to its endogenously formation, AGEs and their precursors are also absorbed from exogenous sources, such as cigarette smoke and through the consumption of highly heated processed foods [3]. Globalization and industrialized methods of processing dramatically alter foods to give desirable properties as longer shelf life, sterility, flavor and color, thus increasing exposure to AGEs [4,5]. AGEs are related to modifying chemical and functional properties of most diverse biological structures and their formation is accelerated in diseases such as diabetes as a result of chronic hyperglycemia and increased oxidative stress [6].

The formation of glycation products or AGEs and their consequences can be elucidated from studies of in vitro glycation of target proteins, such as albumin and lysozyme, using fructose and methylglyoxal (MGO) as glycation agents [7–11]. Fructose is the most common monosaccharide in human diet mostly because of the high-fructose syrup produced by starch, which is usually added to beverages and baked foods; thus, it has specific metabolic characteristics that could potentially contribute to increase body adiposity and insulin resistance [12,13]. In addition to exogenous sources, fructose is endogenously produced as a consequence of activation of the polyol pathway under hyperglycemic conditions and it can be phosphorylated to fructose-6-phosphate, which is broken down into 3-deoxyglucosone; both compounds are powerful glycating agents that enter into the formation of AGEs [14,15]. Reducing sugars not only contribute directly to formation of AGEs, but also lead to the accumulation of dicarbonyl molecules, such as MGO, which is formed from non-enzymatic degradation of endogenous metabolites, mainly glycolytic intermediates. These molecules are very reactive and contribute to formation of AGEs [16,17].

In relation to protein targets for glycation in this study, albumin constitutes about 50% of the proteins present in the plasma of healthy individuals and has a variety of physiological and pharmacological functions [18,19]. Due to its half-life of approximately 21 days and its high concentration, serum albumin is a highly glycation-sensitive plasma protein [20]. It was demonstrated that elevated glycated albumin levels correlated with the severity of coronary artery disease in diabetic patients [21]. Thus, the level of glycated albumin arouses interest in clinical area, related mostly to its use as a short-to-intermediate term biomarker for glycemic control [22]. Lysozymes are important antibacterial defense proteins present at high levels in saliva, nasal secretion, mucus, serum and in the lysosomes of neutrophils and macrophages [23]. This protein affects cell wall peptidoglycans of gram-positive bacteria and catalyzes their degradation [24]. The balance between hydrophilic/hydrophobic surface of lysozyme plays an important role in its catalytic function and glycation process affects this balance decreasing its defense activity [25].

In recent years, saliva has been widely used for diagnosis and monitoring proteins in the body [26,27]. Moreover, studies have analyzed AGEs in saliva of patients, indicating high levels in patients with chronic diseases [28,29]. Younus, Ahmad and Alam [30] verified that amount of AGEs in the saliva of patients with diabetes increases as the history of this disease increases. Therefore, monitoring these AGEs or glycation products can be a powerful tool to delay diseases’ onset and development [31]. However, evidence in the literature around glycated proteins in saliva as biomarkers for diseases may not be so conclusive yet, since according to Khoury et al. [32] salivary fructosamine as biomarker for diabetes may be doubtful. In this way, more studies are needed to elucidate the perspective of glycation products and AGEs in salivary fluid as biomarkers. Regarding biological fluids for the identification of glycation effects and their products, we highlight saliva as an attractive source for its non-invasive, practical and low-cost collection.

Thus, the present study aimed to analyze structural and functional properties of salivary fluid and target proteins albumin and lysozyme after in vitro glycation by fructose and MGO for 21 days incubation, examining fluorescence intensity, ATR-FTIR and protein profiles, as well as protein carbonyl and thiol groups formation. In addition, we investigated whether the activity and protein profile of the main salivary enzyme sAA was affected by fructose and MGO.

## Materials and methods

### Reagents

Reagents of analytical grade were purchased from Sigma-Aldrich (Sigma, St Louis, MO, USA).

### Volunteers

This study was approved by Ethics and Human Research Committee of Federal University of Uberlandia (protocol number: 4.466.521). For this study, saliva samples were collected from twelve healthy individuals, six men and six women, aged between 18 and 40 years. This number of volunteers was determined according to data from the literature involving saliva analysis [33,34]. After fitting inclusion criteria and giving consent, volunteers performed saliva collection. Four individuals performed an extra saliva collection to obtain purified sAA.

### Saliva collection and processing

Saliva collection was performed on a single day in the morning (08:00 to 12:00) to guarantee the same conditions for all volunteers. Each volunteer received a tube for collection, which was carried out at Biochemistry and Molecular Biology Laboratory of Institute of Biotechnology of Federal University of Uberlandia. Saliva of 12 volunteers was collected using the method according to Navazesh [35], without mechanical stimulation in plastic conical tubes Falcon (50 mL). The volunteers were instructed not to eat food or any type of drink for at least 30 minutes before collection. Just before collecting, volunteers rinsed their mouths with distilled water to clean cell debris and then spit approximately 30 mL of saliva inside the tube. After collection, samples were centrifuged at 3000 rpm for 15 min at 4°C to obtain the supernatant, and then stored at -80°C. Subsequently, these frozen samples were lyophilized, weighed, and then again stored at -80°C until analysis.

### Purification of sAA

This method was performed according to Santos et al. [36]. To obtain purified sAA, saliva samples were collected from four individuals, following the same collection protocol explained in previous topic. The saliva collected from all four volunteers (approximately 80 mL) was reunited and centrifuged at 12000 xg for 12 minutes at 4°C. The supernatant was diluted 1:1 (v/v) in 50 mM Tris-HCl pH 8.0 buffer, containing 10 mM EGTA and 10 mM EDTA and 0.2% sodium azide.

To perform ion exchange chromatography, a glass column was used with 9 cm high x 2 cm in diameter, packed with 63 mL of resin Q-Sepharose fast flow. The column was equilibrated with five volumes of Tris-HCl 25 mM pH 8.0, containing 5 mM EGTA and 5 mM EDTA and 0.2% sodium azide. A volume of 160 mL of the diluted saliva sample was applied in the column. The volume collected was dialyzed in 50 mM ammonium bicarbonate, lyophilized, and stored at -80°C for later analysis. Protein concentration was evaluated by Bradford method [37].

### Glycation assay

The glycation assay was performed with all samples diluted in 200 mM phosphate buffer, pH 7.4 containing 0.02% sodium azide and incubated in the dark at 37°C for 21 days with fructose or MGO, according to protocols adapted by Franco et al. [38] and Justino et al. [39]. Samples containing target proteins were incubated with 1.25 M fructose or 53.3 mM MGO (diluted in 200 mM phosphate buffer, pH 7.4 containing 0.02% sodium azide). These concentrations have been used in studies of glycation inhibition by bioactive compounds from natural products, published by our research group [40,41]. We considered maintaining these concentrations in our experiments, even though they are much higher than human physiological levels, to ensure the glycation occurrence in the salivary fluid.

Samples investigated were the following: Bovine serum albumin (BSA) (50 mg/mL), hen egg white lysozyme (10 mg/mL), saliva (50 mg/mL) and sAA (10 mg/mL), all of them, incubated with fructose and MGO (BSA+F, BSA+MGO, LYS+F, LYS+MGO, SAL+F, SAL+MGO, sAA+F and sAA+MGO); These protein concentrations were based on previous studies from our research group [38–41]. Control non-glycated samples without fructose and MGO were incubated under the same conditions, replacing each one with phosphate buffer. After the incubation period, 20% trichloroacetic acid (TCA) was added in the samples and then centrifuged at 10000 xg for 10 minutes. Pellet was resuspended in water. For ATR-FTIR analysis, pellet was lyophilized. Dosage of proteins was measured by Bradford method [37].

### Analysis methods

#### Fluorescence intensity

The fluorescence intensity of samples was measured in a 96-well microplate using a spectrofluorometer with excitation at 350 nm and emission at 420 nm (Perkin-Elmer LS 55, Massachusetts, USA). Measurements of fluorescence intensity in these wavelengths’ values are used to estimate formation of glycation products and AGEs and represent a qualitative measure of damage by glycation [42–44].

#### Attenuated total reflection—Fourier transform infrared spectroscopy (ATR-FTIR)

The analyzes of infrared spectra were acquired using ATR-FTR spectrophotometer Vertex 70 (Bruker Optics, Reinstetten, Germany) coupled to attenuated total reflectance component (ATR). The crystal material in ATR unit used was a diamond disk as an internal reflection element. The spectra of lyophilized samples were recorded in triplicate. Before each sample analysis the air spectrum was used as a background. The spectra were obtained in a room with a temperature between 22–23°C, 4 cm^-1^ resolution and 32 scans were performed. In sample processing, the baseline was corrected and normalized by vector before performing analyzes. The 1800–900 cm^-1^ region of the spectra of all samples was used as input data for multivariate principal component analysis (PCA) technique and vibrational mode areas were calculated from peaks of interest. PCA is a statistical method used to indicate differences between samples. PCA components were analyzed from total variance plot to obtain optimum number of components for datasets. Principal components (PC) scores plots can reveal common clustering of samples in spectra; and loadings plots associated with FTIR spectra provides score plots interpretation. All pre-processing and spectral analysis steps were performed with Origin Pro 9.1 (OriginLab Corporation, Northampton, United States) [45,46].

#### Measurement of carbonyl and thiol groups attached to proteins

For carbonyl measurement, dinitrophenylhydrazine (DNPH) was added to the samples, which were precipitated with 20% TCA, washed with ethanol-ethyl acetate, and dissolved in 6 mol L^-1^ of guanidine hydrochloride [40]. The absorbance values were recorded at 370 nm (VersaMax, Molecular Devices, Menlo Park, CA, USA) and carbonyl content was calculated using a molar absorbance of 22,000 mol L^-1^ cm^-1^; the results were expressed as nmol ratio of reacted DNPH. The content of free thiols was determined in control and glycated samples according to an established method using 5.5’-dithiobisnitrobenzoic acid (DTNB) [47]. Concentration of free thiol was calculated using 14,150 mol L^-1^ cm^-1^ as molar extinction coefficient of NTNB. Reading at 412 nm was performed using a spectrophotometer.

#### SDS polyacrylamide gel electrophoresis (SDS-PAGE)

The samples after glycation were solubilized in electrophoresis buffer (1:10) (Tris-HCl 31.2 mM, SDS 8.75%, sucrose 20%, β-mercaptoethanol 10%, EGTA-K 11 mM and bromophenol blue 0.25%), and then submitted to electrophoresis in sodium dodecyl sulfate (SDS)—polyacrylamide gel (SDS-PAGE) using 12% polyacrylamide gels for analysis of protein profile [48]. Samples containing 5 μg of proteins [37] were loaded into the wells and electrophoresis occurred at 35 mA (Bio-Rad Laboratories, Hercules, USA). The gels were stained with Coomassie blue R, destained with solution of methanol-acetic acid-water (25:7:68) and photographed by digitalization (Amersham Imager 600 system, GE Healthcare Bio-Sciences AB, Uppsala, Sweden).

#### Western blotting

Aliquots of saliva samples were solubilized in electrophoresis sample buffer. 5 μg of proteins from each sample were submitted to SDS-PAGE. The samples separated by SDS-PAGE were transferred to a nitrocellulose membrane in Tris-glycine buffer [49]. After transferred, membranes were blocked with 5% skimmed-milk powder in PBS-Tween 20 (PBS-T) and washed 3 times (2 times for 5 minutes and 1 time for 10 minutes) with PBS-T. After being blocked and washed, membranes were incubated overnight with primary anti-amylase antibody [36] followed by washing (twice for 5 minutes and once for 10 minutes) with PBS-T. The membranes were incubated with secondary anti-rabbit antibody (GE Healthcare Bio-Sciences AB, Uppsala, Sweden), and washed with PBS-T. The antibodies bounded to the membranes were visualized by chemiluminescence. Intensity of proteins bands was analyzed by ImageQuantTL software, and results were expressed as densitometry of pixels.

#### sAA activity

To determine sAA activity after glycation, a colorimetric kinetic assay was used. This method is based on the hydrolysis of substrate 2-chloro-4-nitrophenyl-4-β-D galactopyranosylmaltoside (GalG2CNP), by alpha-amylase, releasing 2-chloro-4-nitrophenyl (CNP). sAA samples were diluted in MES buffer (MES 50 mM, NaCl 300 mM, CaCl_2_ 5 mM, KSCN 140 mM, pH 6.3), followed by addition of GaLG2CNP substrate and reading was performed on a spectrophotometer, for 3 minutes at 37°C, with interval of 1 minute between each reading [50].

### Statistical analysis

Statistical and graphical analyzes were performed using GraphPad Prism 6.0 software. All analyzes were performed in duplicate and data were expressed as mean ± standard deviation. The significance of difference was calculated using one-way or two-way ANOVA, and Tukey and Dunnett’s post-tests for multiple comparisons. Values of p <0.05 were considered significant.

## Results

### Fluorescence intensity

**Fig 1:**
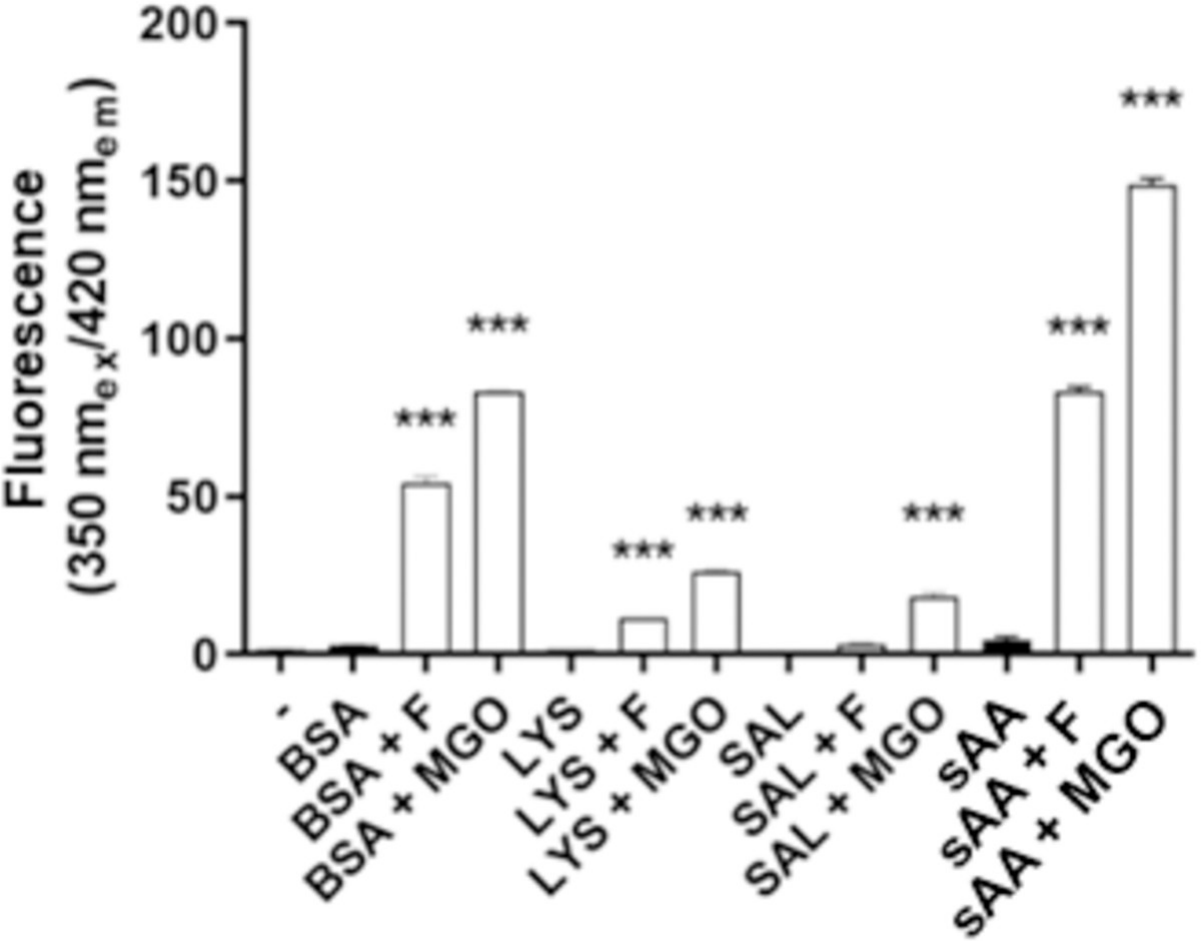
Fluorescence intensity of non-glycated BSA, LYS, SAL and sAA, and glycated by fructose or MGO. *** p < 0,001.

The fluorescence intensity of BSA, lysozyme, saliva and sAA when incubated for 21 days with fructose or MGO is shown in Fig 1. Fluorescence increased in glycated samples by fructose and MGO when compared to non-glycated samples. Values of these increases were as follows: BSA+F (51.6) and BSA+MGO (80.5); LYS+F (9.7), LYS+MGO (24.8); SAL+MGO (17.0); sAA+F (80.0) and sAA+MGO (148.8). However, saliva incubated with fructose showed no difference. Fluorescence intensity of the samples after 7 days of incubation is available as Supporting Information (S1 Fig).

### ATR-FTIR

In order to evaluate spectral differences related to glycation process with fructose and MGO after incubation of 21 days, spectra of samples were submitted to PCA analysis. A clear separation between glycated BSA and lysozyme were observed in the score plots. The percentage of variance explained by the first two PCs showed 99.9% for BSA+F and 100% for BSA+MGO, LYS+F and LYS+MGO (Supporting Information, S2A, S2C, S3A and S3C Figs). Saliva PCA score plot showed an efficient separation of 83.9% by variance explained with the first two PCs of SAL+F, and a moderate distinction of 74.7% of SAL+MGO (Supporting Information S4A and S4C Fig). The graph shows that the first two PCs separate samples data into two clusters.

**Fig 2:**
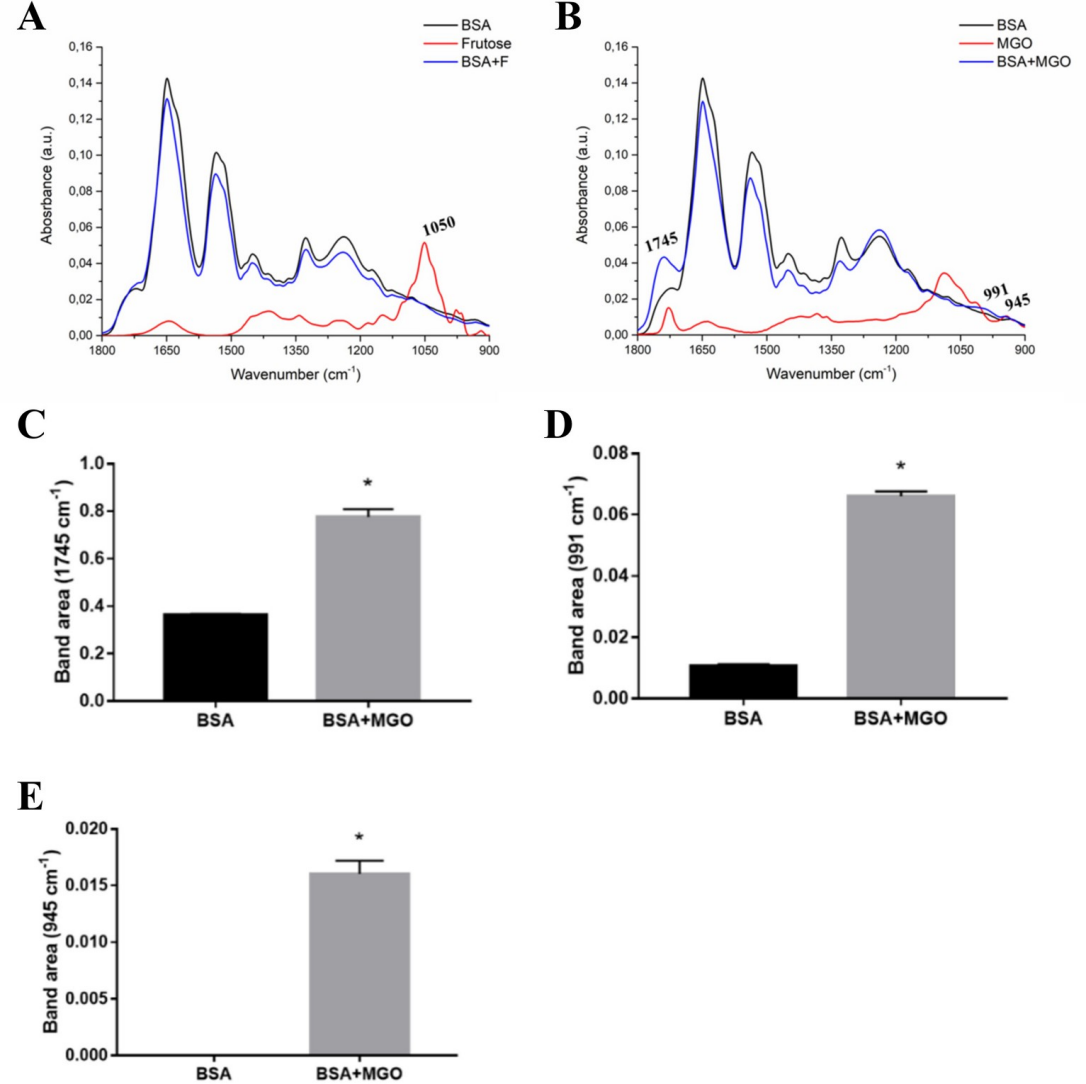
Average ATR-FTIR spectra (1800–900 cm^-1^) of BSA, fructose, MGO, and BSA incubated with these glycating agents. (A) BSA and BSA with fructose (BSA+F). (B) BSA and BSA with MGO (BSA+MGO). Peak area of the spectra in vibrational modes in 1745 cm^-1^ (C), 991 cm^-1^ (D) and 945 cm^-1^ (E).

**Fig 3:**
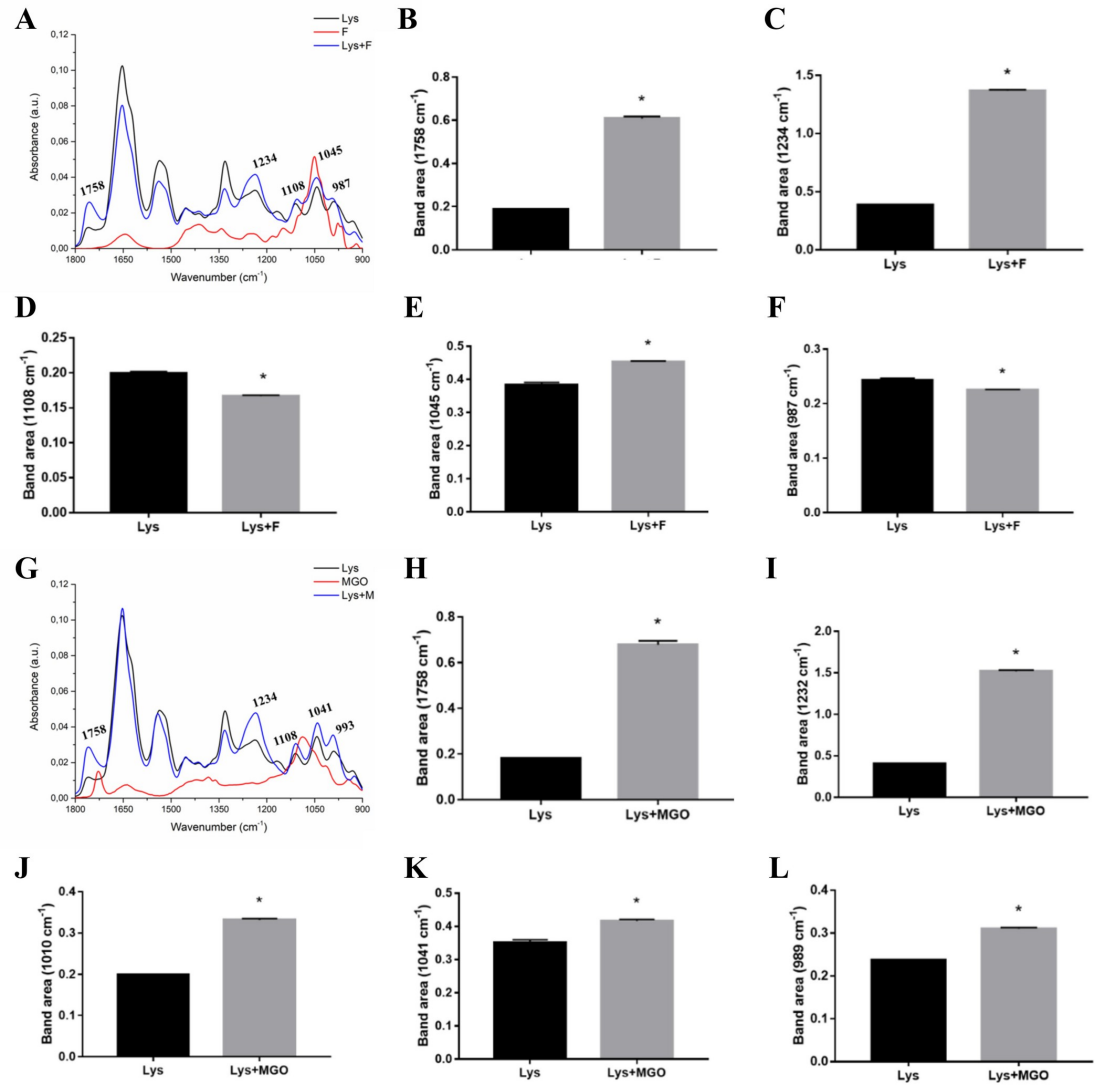
Average ATR-FTIR spectra (1800–900 cm^-1^) of LYS, fructose, MGO, and LYS incubated with these glycating agents. (A) LYS and LYS with fructose (LYS+F). Peak area of the spectra in vibrational modes in 1758 cm^-1^ (B), 1234 cm^-1^ (C), 1108 cm^-1^ (D), 1045 cm^-1^ (E) and 987 cm^-1^ (F). (G) Average spectra of LYS and LYS with MGO (LYS+MGO). Peak area of the spectra in vibrational modes in 1758 cm^-1^ (H), 1234 cm^-1^ (I), 1108 cm^-1^ (J), 1041 cm^-1^ (K) and 993 cm^-1^ (L).

**Fig 4:**
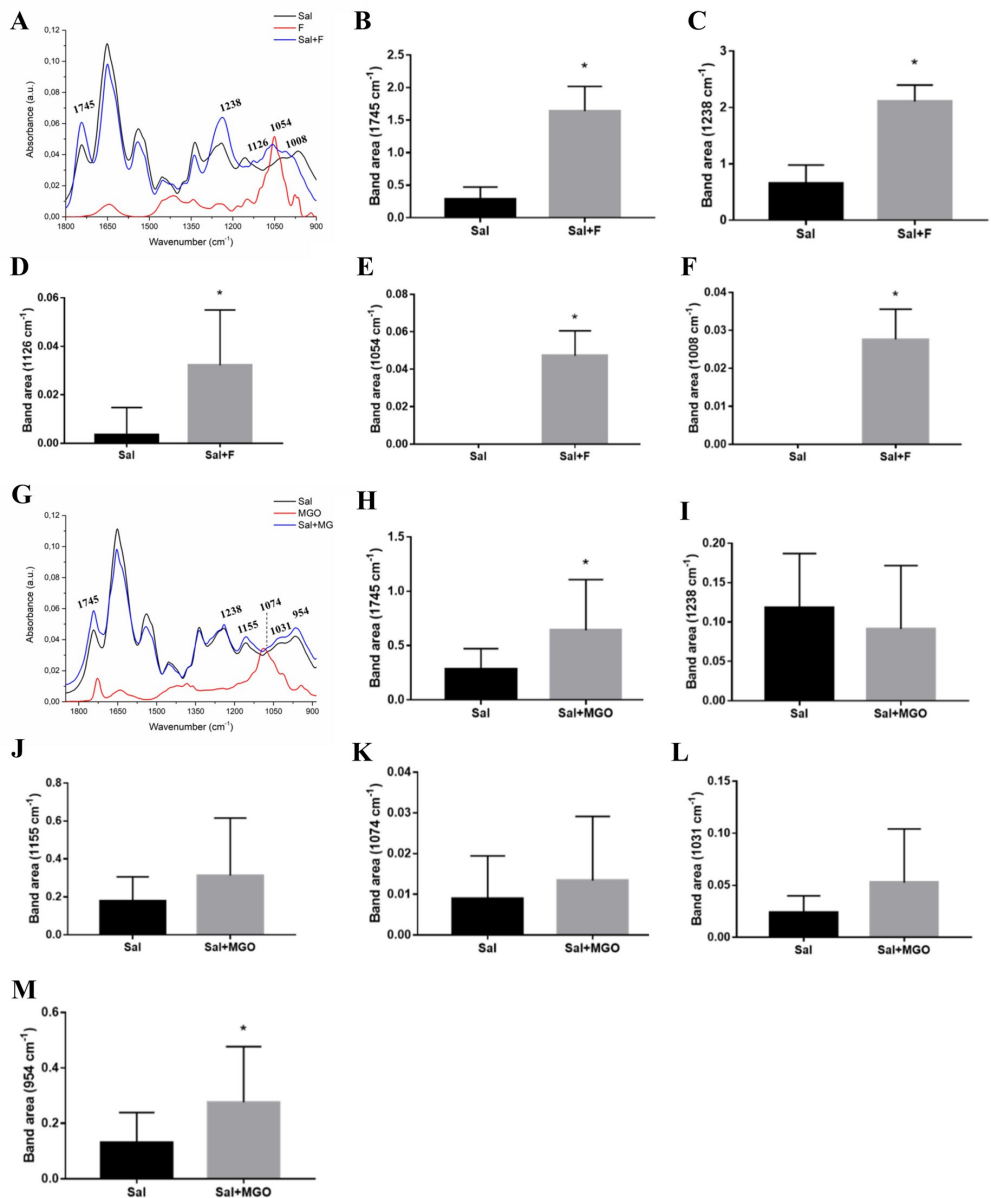
Average ATR-FTIR spectra (1800–900 cm^-1^) of SAL, fructose, MGO, and SAL incubated with these glycating agents. (A) SAL and SAL with fructose (SAL+F). Peak area of the spectra in vibrational modes in 1745 cm^-1^ (B), 1238 cm^-1^ (C), 1126 cm^-1^ (D), 1054 cm^-1^ (E) and 1008 cm^-1^ (F). (G) Average spectra of SAL and SAL with MGO (SAL+MGO). Peak area of the spectra in vibrational modes in 1745 cm^-1^ (H), 1238 cm^-1^ (I), 1155 cm^-1^ (J), 1074 cm^-1^ (K), 1031 cm^-1^ (L) and 954 cm^-1^ (M).

To evaluate the relation between PCs and original variables, loadings graph shows how the original variables report to PCs (Supporting Information, S2B–S4B and S2D–S4D Figs). In this way, ATR-FTIR data followed by PCA analysis could be used to indicate general changes in glycation process caused by incubation for 21 days with fructose and MGO, without necessarily identifying specific vibrational modes of glycation process. With this, spectral profile of fructose and MGO and samples of BSA, lysozyme and saliva incubated with these glycation agents are represented in Figs 2A, 2B, 3A, 3G, 4A and 4G. From the peak information shown by graphs of PCA loads and spectra of fructose and MGO agents, it was possible to visualize peaks of interest that represented glycation process.

The main highlight of fructose spectrum was the 1050 cm^-1^ vibrational mode (OH and CH2). The comparison of BSA spectra did not show any specific differences in glycation process between BSA and BSA+F (Fig 2A). Glycation of BSA with MGO, on the other hand, showed important differences in the spectrum (Fig 2B). An increase in peak areas in vibrational modes were observed at 1745 cm^-1^ (C = O stretching vibration of proteins and polysaccharides), 991 cm^-1^ (COC and CO by ring vibrations of carbohydrates; COH bonds and OCH3 of polysaccharides) and 945 cm^-1^ (COC stretching and OH, COC deformation of carbohydrates) (Fig 2C–2E, respectively).

The spectra of lysozyme, fructose and lysozyme incubated with fructose showed characteristic differences as indicated in Fig 3A, highlighting the wavelengths of different peaks. Analysis of the area of these peaks shows respective area increases in 1758 cm^-1^ (C = O of the polysaccharides), 1234 cm^-1^ (components of the CN amide III band of proteins) and 1045 cm^-1^ (CO elongation frequencies coupled to CO flexion frequencies of the C-OH groups of carbohydrates) (Fig 3B, 3C and 3E). However, there was a decrease in peak areas in 1108 cm^-1^ (COOH lengthening of the sugar portions) and 987 cm^-1^ (C-O-C, C-O of carbohydrates and OCH3 of polysaccharides) (Fig 3D and 3F). Regarding the effect of incubation with MGO on glycation of lysozyme, it was possible to observe marked changes in the spectrum (Fig 3G). Analysis of the area of peaks modified by lysozyme glycation shows an increase in 1758 cm^-1^, 1234 cm^-1^, 1108 cm^-1^, 1041 cm^-1^ (CO elongation frequencies coupled with CO fold frequencies of groups C- OH of carbohydrates) and 993 cm^-1^ (COC, CO of carbohydrates and OCH3 of polysaccharides) (Fig 3H–3L). As seen in BSA glycation spectrum, these vibrational modes represent structural components mainly related to proteins, polysaccharides and carbohydrates.

Fig 4A represents the average spectra of saliva, fructose, MGO and glycated saliva after incubation with these agents. The main peaks evaluated for fructose-glycated saliva showed several differences in the spectrum with an increase in peak band area at 1745 cm^-1^ (C = O stretching vibration of proteins and polysaccharides), 1238 cm^-1^ (CN amide III band components of proteins, CO stretching in carboxylic acid and PO2- stretching), 1126 cm^-1^ (CO of carbohydrates), 1054 cm^-1^ (oligosaccharide C-OH bonds) and 1008 cm^-1^ (CH2OH groups, CO stretching and COH groups bending) (Fig 4B–4F). It is important to highlight that peaks 1054 cm^-1^ and 1008 cm^-1^ were exclusive to the incubation of saliva with fructose, therefore did not found in non-glycated saliva. Saliva and MGO spectra is represented in Fig 4G. However, there was observed an increase only in area of peaks bands at 1745 cm^-1^ and 954 cm^-1^ (C-O-C, C-O of carbohydrates) of SAL+MGO (Fig 4H and 4M, respectively).

### Levels of carbonylated protein and thiol groups

**Fig 5:**
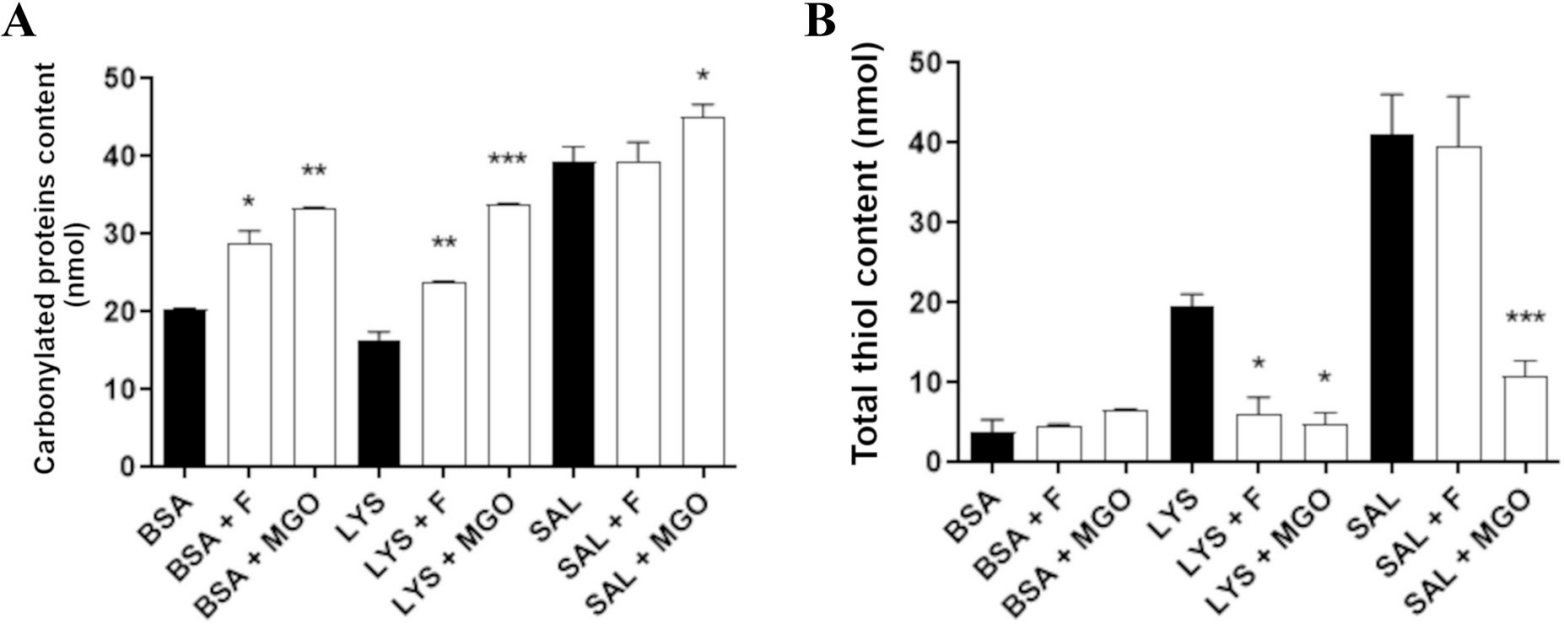
Carbonylated proteins (A) and total thiol contents (B) in non-glycated BSA, lysozyme and saliva, and glycated by fructose or MGO. * p < 0,05; ** p < 0,01; *** p < 0,001.

The levels of carbonylated proteins and total thiols in the target proteins and salivary fluid are shown in Fig 5. Protein carbonylation (Fig 5A) was increased in BSA+F, BSA+MGO, LYS+F, LYS+MGO and SAL+MGO, while SAL+F was not different from non-glycated saliva. Total thiols levels in BSA, lysozyme and saliva are shown in Fig 5B. For BSA, there was no significant difference of thiols level among glycated (fructose and MGO) to non-glycated BSA. On the other hand, comparing with non-glycated lysozyme, thiols levels decreased for LYS+F and LYS+MGO, while for saliva, only SAL+MGO showed a reduction in thiols content.

### Protein profile by SDS-PAGE and immunodetection

**Fig 6:**
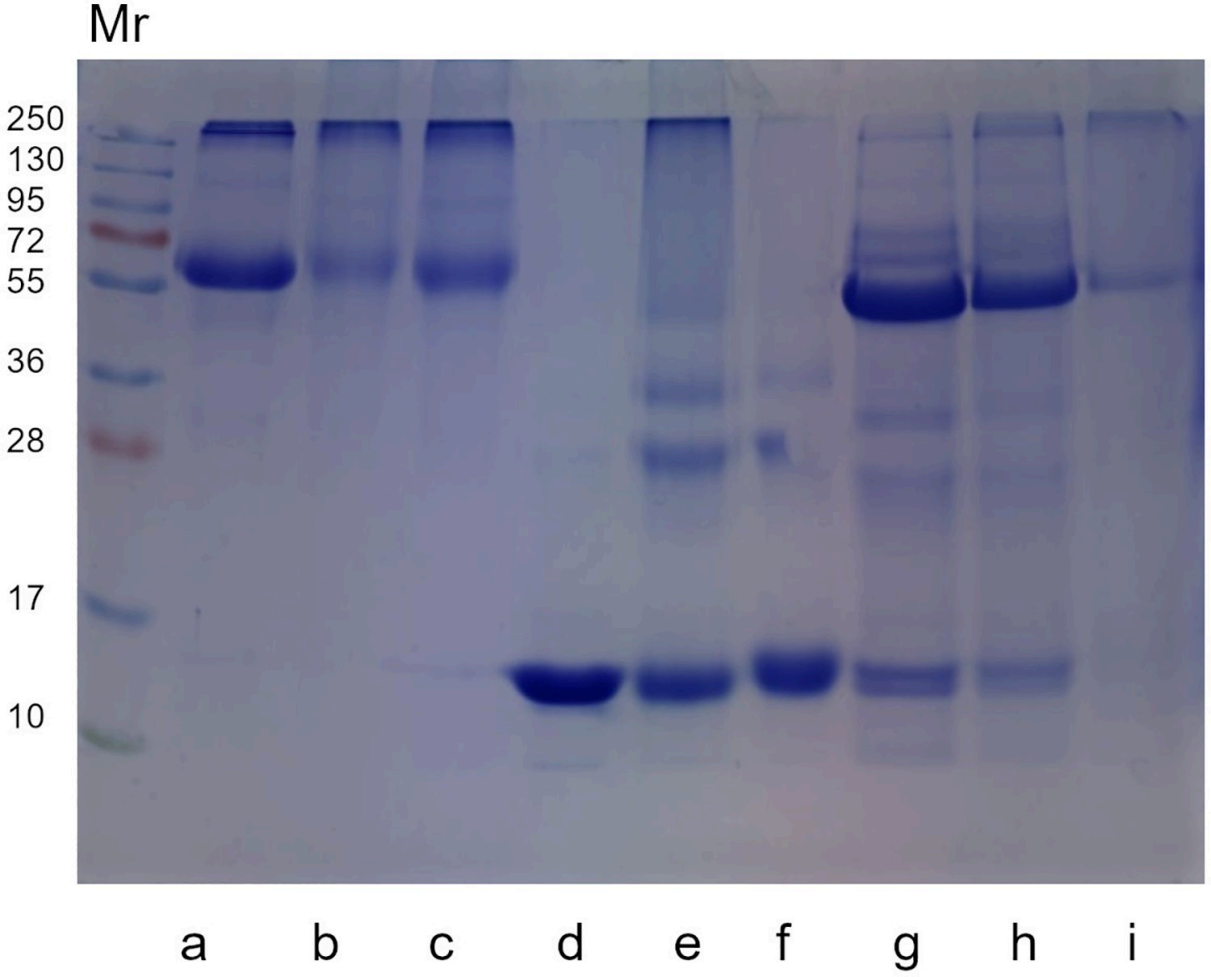
Protein profile (SDS-PAGE) of non-glycated BSA, LYS and saliva and glycated by fructose or MGO. Each lane was loaded with the following sample: (a) BSA; (b) BSA+F; (c) BSA+MGO; (d) LYS; (e) LYS+F; (f) LYS+MGO; (g) SAL; (h) SAL+F; (i) SAL+MGO. Mr: Relative molecular mass of the protein standard (kDa).

Protein profile in SDS-PAGE (Fig 6) of non-glycated and glycated BSA, LYS and SAL suggest the formation of crosslinks in glycated proteins due to changes in intensity of bands’ staining or appearance of new bands after glycation. BSA (66kDa) reduced Mr 66 band staining when glycated with fructose and MGO compared to non-glycated BSA. In addition, it is possible to detect a possible new band in Mr 95 of glycated BSA. In LYS, (14 kDa) Mr 14 band is intensely colored. However, its intensity reduced in LYS+F and LYS+MGO. Moreover, bands of higher Mr are more observed in LYS+F suggesting the formation of oligomers (28, 36 and 55 kDa). Protein profile of non-glycated saliva reveals two prominent bands with different Mr, we highlight Mr 56 and Mr 14 corresponding to sAA and lysozyme respectively. Glycation of saliva caused a reduction in staining intensity of these bands, also suggesting formation of crosslinks that may made it difficult to enter the polyacrylamide gel. Immunodetection was performed with saliva samples and complements the result by showing the effect of glycation on the expression of Mr 56 band. In SAL+MGO there was a reduction of approximately 74% in pixel intensity of this band (S5A and S5B Fig). In Supporting Information, protein profile of all saliva and sAA samples can be found after 21 days of incubation (S6A–S6C and S7 Figs, respectively), as well as the protein profile of sAA after 7 days of incubation (S8 Fig), and image of all blot (S1 Raw images).

### Purified sAA activity

**Fig 7:**
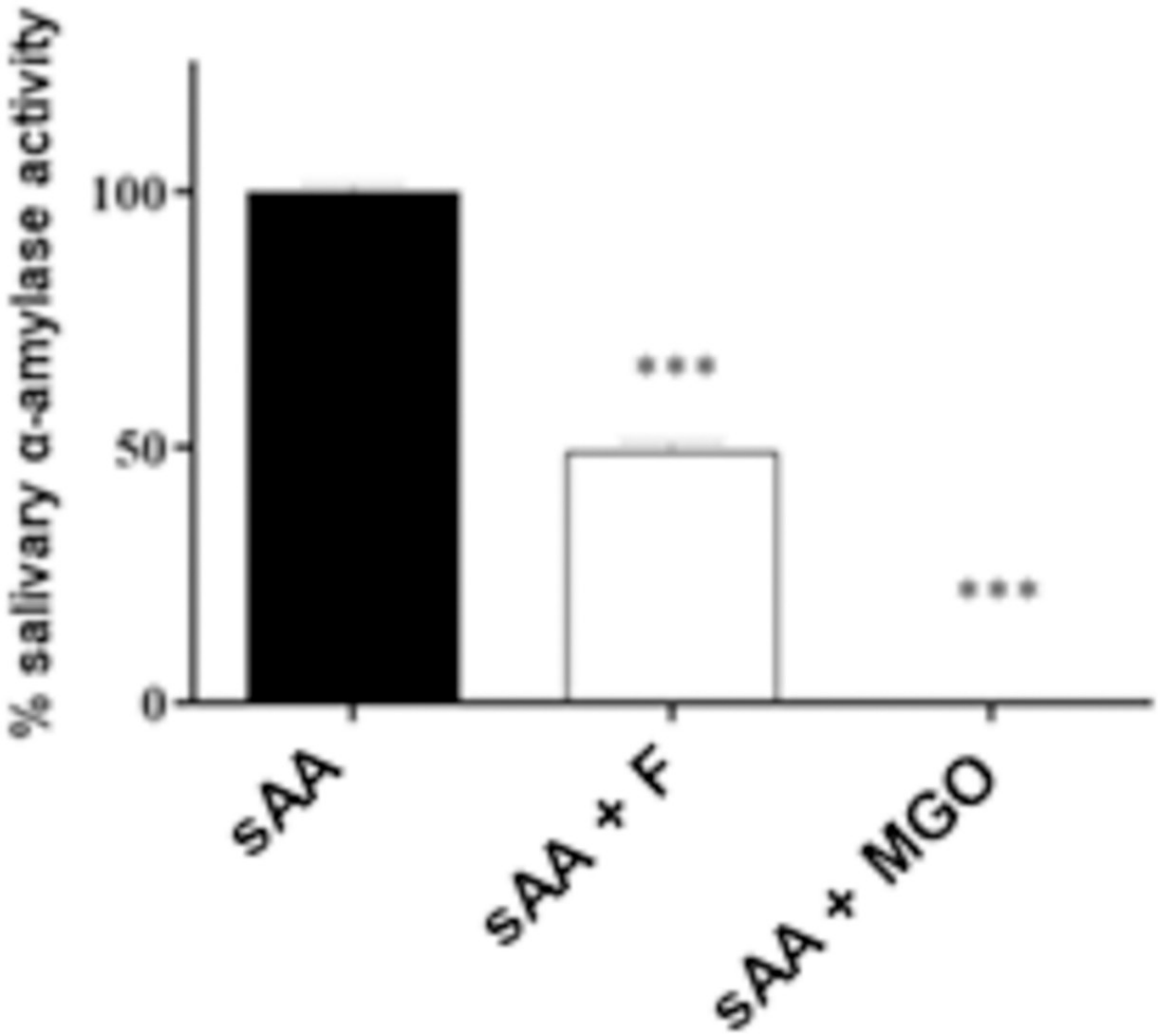
Enzymatic activity of non-glycated sAA, and glycated by fructose or MGO. *** p < 0,001.

Purified sAA was evaluated for its enzymatic activity, as shown in Fig 7. The result indicates significant decreases of this enzyme activity. sAA+F presented 49% of activity in relation to non-glycated sAA, and sAA+MGO reveled drastic reduction of activity with only 0.2% in relation to non-glycated sAA. sAA activity after 7 days of incubation is shown in Supporting Information (S9 Fig).

## Discussion

The study of proteins containing early-stage glycation products or AGEs has become of great interest due to evidence of AGEs effects on protein function, tissue damage and oxidative stress in aging and some diseases specially diabetes [50–55]. In this study, salivary fluid and proteins albumin, lysozyme and sAA were used as targets for non-enzymatic glycation by fructose and MGO for 21 days.

Levels of glycation products or AGEs depend on the half-life of proteins. On long-lived proteins they even accumulate over the lifetime of organisms [56]. In general, circulating proteins have a relatively short half-lives compared to structural proteins [57]. Some of the proteins present in saliva have short half-lives such as albumin with approximately 15–20 days [58]; lysozyme with 7 days [59]; immunoglobulin A with 3–6 days, plasmatic amylase has a half-life around 12–24 hours [60], however, in saliva the data seem to be not so clear yet. For having shorts half-lives, these proteins may underestimate the accumulation of AGEs [57] but could represent the glycation status more precisely in the last few days or hours. According to the proteins half-lives above, incubation period of 21 days in our study was enough for proteins to be glycated.

The concentrations of glycation agents used in our incubation were based on previous studies of our research group about antiglycation properties of natural products [40,41]. They are much higher than physiological levels, which range around 35 μM for fructose and 10 μM for MGO. These low values make hard to translate them into a good circulating marker of AGEs [61,62]. Kinetically, fructose may have a very fast conversion of Heyns compounds so that an increased fructose intake from the diet might potentiate the Maillard reaction [63]. Thereby AGEs formed in the intestinal lumen from the diet may contribute to the circulating AGE levels [61]. Although the concentrations of fructose and MGO were high, they might be convenient for modeling the glycation process that occurs in the organism in physiological condition in short time over weeks or months [64]. For ensuring the glycation occurrence in the salivary fluid, we maintained those high concentrations so that it was possible to visualize the effects of glycation, since saliva in vitro glycation is not very approached in literature.

Based on fluorescence properties it was possible to observe an increase in fluorescence intensity of glycated proteins in this study. Analyzing glycated albumin by fructose (BSA+F), we found corresponding results in other studies [65,66]. It has been shown that early and advanced glycation of BSA by fructose has a site specificity, with lysine-524 residue being the main target for BSA modification. Almost every change of lysine in glycated BSA with fructose was attributed to formation of carboxymethylysine (CML) [9]. On the other hand, the effect on albumin exposed to MGO caused a greater increase in fluorescence intensity, which is in line with other results [67,68]. The irreversible changes generated by glycation of MGO in BSA are highly selective for arginine residues, with some modifications of lysine and amino-terminal groups [69,70]. Many studies show that high concentrations of glycated albumin are associated with several diabetic complications, and this has been reported as a powerful indicator of glycemic control because its half-life is shorter than that of HbA1c, better representing glycemic variations [71,72].

Fluorescence intensities in glycation of lysozyme by fructose and MGO were also increased, which coincides with previously found results [11,73]. Glycation process of lysozyme results in reduction of its antibacterial function. Such changes are an important factor in increasing the prevalence of bacterial infections observed in patients with diabetes [23]. MGO is highly reactive and reacts with lysozyme at a higher rate than glucose, resulting in formation of crosslinked AGEs [74].

Glycated saliva by fructose showed no changes on fluorescence intensity. One possible reason for this result is related to saliva antioxidant capacity. It was demonstrated that AGE fluorescence was significantly lower in healthy adults, in response to the most effective antioxidant defense in people aged 25–45 [75]. Moreover, salivary proteins have several functions besides digestive. There are many enzymes with important properties for oral health, regulation of defense and endocrine systems [76]. Thus, it is possible that these enzymatic activities in saliva may also influenced the glycation state indicated by fluorescence. Otherwise, effect of saliva glycation on fluorescence intensity generated a significant increase in SAL+MGO. It is known that MGO forms hydroimidazolone derived from modification of arginine (MG-H1) and also carboxyethylisine (CEL) [53]. Manig et al. [77] suggested that levels of glycation compounds in saliva may be useful biomarkers to assess diabetes, since some AGEs found in saliva, such as CEL, CML and MG-H1, are higher in diabetic individuals. Studies involving the formation of AGEs in a hyperglycemic state have shown that MGO is considered the main precursor to AGEs, which can lead to enzymatic inactivation and protein denaturation [7,78]. A recent study corroborates our data showing high levels of AGEs associated with oxidative stress biomarker of protein oxidation and lipid peroxidation in saliva of patients with chronic heart failure [28].

Results of sAA showed a significant increase in fluorescence intensity due to glycation promoted by incubation with fructose and MGO. This higher fluorescence of glycated sAA may be related to the reduction of its activity as observed in glycated sAA by fructose and MGO. Several studies that investigated levels of sAA in diabetics and healthy individuals, resulted in reduction of their activity and concentration in diabetics, corroborating our result [79–81]. Also, Klimiuk et al. [28] reported a reduction in sAA activity correlated with an increase of AGEs in saliva of patients with progressive chronic heart failure.

As a method used to analyze structural changes in molecules, ATR-FTIR spectroscopy clearly indicated changes in the structure of albumin, lysozyme and saliva caused by glycation by both fructose and MGO. For sAA analysis, methods of sample preparation are still being refined for better readings in the equipment.

In glycation process molecules are attached to albumin at multiple side chains, like lysine, arginine and cysteine residues that induce secondary and tertiary structure changes, contributing to alterations in biological and physiological properties of this protein, such as drug and metabolite binding capacity and free radical scavenging. These post-translational modifications of glycated albumin may be related to long term diabetic complications [20,82,83].

Lysozyme is a protein consisting of ~40% of the α-helical structure and has six lysine residues and eleven arginine residues as potential glycation sites [84,85]. Studies previously showed that glycation alters conformation of lysozyme secondary and tertiary structures, and the loss of α-helix results in reduction in its bactericidal and enzymatic activity, thereby increasing susceptibility to bacterial infections in diabetes [86–88].

In some diseases such as diabetes and hypertension, saliva presents changes in its protein components, and glycation process may contribute to impair their proteins functions [23,89]. Ansari et al. [90] used FTIR technique to verify Amadori’s products and concluded that glycated protein can be a good diagnostic biomarker for early glycation process in diabetes. These possible biomarkers may exhibit specific signatures in infrared spectrum of saliva, as vibration absorbances of side chain spectra associated with glycation appear to be quite prominent in this fluid [91].

Vibrational mode regions can be slip up so that amide I is represented by region around 1600-1800cm^−1^, amide II around 1400–1500 cm^−1^, amide III around 1200–1400 cm^−1^ and at lower wavenumbers, amino acid and sugar molecules between 750–1100 cm^−1^ [85]. Glycation process in this study contributed to increase intensity of unique vibrational modes. We emphasize the difference in vibrational modes at ~1750 cm^-1^ and ~1050 cm^-1^, which are related to carbonyl functional groups and carbohydrate/sugar peak vibrations, respectively. These findings are in line with glycation reaction process, which involves reducing sugars, in polyol pathway with free aldehyde and an amine group, usually in a side chain of lysine and arginine [92,93].

Thus, these data suggest that vibrational modes at 1745 cm^-1^ and 1758 cm^-1^ (C = O of carbonyl group) can be used to distinguish the presence of glycation in these proteins. The formation of new molecules detected by ATR-FTIR may be related to aldehyde terminal group in glycation process [94]. Therefore, data presented by ATR-FTIR spectroscopy confirmed changes in glycated proteins both when exposed to fructose or MGO for 21 days. The exception was albumin sample incubated with fructose which it was not possible to detect these changes.

Protein carbonylation can be provoked through covalent modification of proteins with oxidative by-products of reducing sugars, such as reactive dicarbonyl molecules MGO, glyoxal and 3-deoxyglucosone [17,52,95]. Glycation of albumin and lysozyme by fructose and MGO generated a significant increase in carbonyl content, which agrees with results found in previous studies [96–98]. A study that quantified the formation of carbonylated protein in saliva indicated higher levels in diabetics, corroborating our result of an increased level of carbonylation when incubated with MGO in saliva [99]. Carbonyl groups are indirectly introduced into proteins by covalent adduction of reactive carbonyl species to side chains of the nucleophilic amino acids arginine, lysine and cysteine [100]. Interaction of proteins with reactive carbonyls can result in inactivation and modification of essential cellular proteins that can potentially lead to cytotoxicity and contribute to diseases, as indicated in a study that detected elevated amount of protein carbonyls in cardiovascular disease [101].

Dicarbonyl compounds also react rapidly with thiol groups of amino acids, peptides and proteins to give thiol-aldehyde adducts at cysteine residues and this depletion may enhance oxidative damage in proteins [102]. The results of free thiols showed a reduction in their levels for glycated lysozyme by both fructose and MGO. Whereas for saliva only MGO reduced thiol content, indicating association between glycation products and oxidative state of proteins. Studies corroborate these results showing that glycation process provoked a reduction in free thiol group of proteins [83,103,104]. Moreover, Rajeshwari et al. [105] found decreased levels of thiols in saliva of diabetic group in relation to healthy individuals. Thiols are able to scavenge oxidants playing a significant role in protecting biomolecules from oxidative stress [106,107]. Thus, their glycation by carbonyl compounds is highly associated with complications of diseases such as diabetes, in which oxidative and carbonyl stress are elevated leading to decrease in free thiol contents and antioxidant capacity [54,108,109]. sAA represents the most abundant protein in salivary fluid [110], then, we presume that both results of carbonylation and thiol content in glycated saliva would represent sAA as well.

From analysis of SDS-PAGE, it was possible to observe in glycated albumin that a modification of its polypeptide chain of 66 kDa occurs, losing its intensity. Alqahtani et al. [111] also found in their study that MGO had an influence on the electrophoretic pattern of BSA which resulted in lower intensiveness protein band compared to untreated protein. Besides, the appearance of a subtle but distinct band when compared to profile of non-glycated albumin suggested a possible correspondence with a tripolymer that emerged as an indication of crosslinking in glycated albumin. This type of observation has also been described in Zhang et al. [112]. The electrophoretic profile of glycated lysozyme also showed formation of high molecular weight products in the presence of fructose and MGO, characterizing crosslinked lysozyme. These products represent dimers, trimers, and tetramers with molecular weights of approximately 28, 36 and 55 kDa, respectively, induced by glycation process [113]. This corroborates our previous results that showed occurrence of glycation in lysozyme. In electrophoresis of saliva samples, all bands had their intensity decreased with glycation by fructose and MGO, however, formation of probable bands was not observed that would confirm the crosslinking of proteins. One possibility that explains this result is the great heterogeneity of compounds present in salivary fluid, hindering the specific detection of AGEs. There is lack of studies in literature that verify by SDS-PAGE the formation of glycation products or AGEs in glycated saliva, therefore, more researches are needed in this area.

Finally, saliva samples were also subjected to Western blotting, which revealed a significant reduction in expression of sAA, after glycation with MGO. This corroborates previous results in this study, in which saliva showed increased fluorescence intensity and oxidative damage by MGO, as well as difference in vibrational modes in ATR-FTIR, confirming alterations by glycation process. Besides, glycation generated accentuated reduction of sAA activity, especially after MGO incubation. These results indicate that glycation process caused structural alterations on sAA, which contributed for reduction of its enzymatic activity and consequently decrease their band expression. Lower sAA activity has been correlated with oxidation and protein glycation products and several studies showed significant decrease of sAA activity in diabetes [79,80,114,115]. In addition, according to Mekahli et al. [116], hyperglycemia induces perturbation in the intracellular Ca^2+^ signaling in salivary glands and leads to improper post-translational processing and folding of proteins, consequently decreasing sAA activity in diabetic individuals.

Among all analysis methods performed in this study, we highlight the fluorescence as the most sensible, significative, and practical for using in samples of patients. According to Perrone et al. [31], estimation of serum, urine, and saliva AGEs might be measured by spectroscopic and fluorimetric methods. Pentosidine is one of the fluorescent AGEs and it is a well-accepted marker of cumulative protein damage in aging and a variety of disease states including diabetes [117]. However, the amount of AGEs quantified by this method provides only the quantification of fluorescent AGEs and may be interfered by non-AGE fluorophores [31].

Given all the results obtained in our study, we showed that it is possible to identify glycation products in salivary fluid, and its applicability may contribute to the area of biomarkers in diseases. However, this is a field that still needs to be well investigated, deepened, and should be treated with caution, as studies have also shown that there might not be such a conclusive relation between AGEs, saliva and diagnosis. Yilmaz et al. [118] found a strong correlation between salivary AGEs and HbA1c, otherwise Rao et al. [119] showed weak correlation between salivary protein glycosylation and HbA1c. If salivary fluid is really remarkable to correlate AGEs specifically in diseases, this fluid will be a very promising tool for the diagnosis and monitoring the glycation process, its products and its consequences in various diseases.

The findings of this work promoted the understanding around structural and functional alterations of proteins submitted to glycation by fructose and MGO. In vitro glycations of BSA and hen egg white lysozyme, both homologous to human proteins [120,121], are well established in the literature and could be used as glycation models in order to analyze and improve in vitro glycation assay in saliva, which has become in recent years a promising fluid in clinical area to be used as an indicator of glycation process and salivary protein alterations present in several diseases. We believe that our method configurates a previous vision around glycation products in saliva by biochemical and spectroscopic approach. Adjustments of protein and glycation agents’ concentrations are needed for better representation; or further, analysis of samples in real specific conditions of diseases, until we get a certain conclusion about biomarkers as AGEs or glycation products in salivary fluid for diagnosis.

## Conclusion

Overall, in this study glycated samples presented increased florescence intensity, structural changes related to vibrational modes of protein glycation, increased protein carbonylation, decreased thiol groups, protein profile alterations and relative reduction of band corresponding to Mr of sAA and its activity. These results indicate that fructose and/or MGO lead to formation of glycation products, protein alterations and oxidative damage when incubated with saliva, BSA and lysozyme for 21 days. Plus, sAA had reduction of activity and alterations of protein profile as well. We emphasize saliva as a possible biological fluid for glycation analysis. Thus, glycation products and AGEs in saliva may have the potential to contribute to a possible improvement in assessment and monitoring of patients with diseases in an accessible and non-invasive way. In addition, due to scarce studies in literature involving in vitro glycation of saliva and sAA, this study may contribute to direct more research and open perspectives in this area.

## Supporting information

S1 Fig Fluorescence intensity of non-glycated BSA, LYS and SAL and glycated by fructose or MGO after 7 days incubated. ** p < 0,01; *** p < 0,001.
(TIF)

S2 Fig Principal component analysis (PCA) plots of BSA. (A) Scores scatter plot of PC1 vs PC2 performed on the BSA and BSA+F spectrum. (B) PC1 loadings profile obtained for BSA and BSA+F. (C) Scores scatter plot of PC1 vs PC2 performed on the BSA and BSA+MGO spectrum. (D) PC1 loadings profile obtained for BSA and BSA+MGO.
(TIF)

S3 Fig Principal component analysis (PCA) plots of LYS. (A) Scores scatter plot of PC1 vs PC2 performed on the LYS and LYS+F spectrum. (B) PC1 loadings profile obtained for LYS and LYS+F. (C) Scores scatter plot of PC1 vs PC2 performed on the LYS and LYS+MGO spectrum. (D) PC1 loadings profile obtained for LYS and LYS+MGO.
(TIF)

S4 Fig Principal component analysis (PCA) plots of SAL. (A) Scores scatter plot of PC1 vs PC2 performed on the SAL and SAL+F spectrum. (B) PC1 loadings profile obtained for SAL and SAL+F. (C) Scores scatter plot of PC1 vs PC2 performed on the SAL and SAL+MGO spectrum. (D) PC1 loadings profile obtained for SAL and SAL+MGO.
(TIF)

S5 Fig Immunodetection of non-glycated sAA and glycated sAA by fructose or MGO. (A) Western blotting of sAA expression in SAL (lane a), SAL+F (lane b), and SAL+MGO (lane c). (B) Quantification of immunodetected sAA given in density of pixels.
(TIF)

S6 Fig Protein profile (SDS-PAGE) of non-glycated saliva and glycated saliva by fructose or MGO after 21 days. (A,B and C) Each lane was loaded with de following sample: (a): SAL; (b): SAL+F; (c): SAL+MGO; Mr: relative molecular mass of the protein standard (kDa).
(TIF)

S7 Fig Protein profile (SDS-PAGE) of non-glycated sAA and glycated sAA by fructose or MGO after 21 days. Each lane was loaded with de following sample: (a): sAA (b): sAA+F; (c): sAA +MGO.
(TIF)

S8 Fig Protein profile (SDS-PAGE) of non-glycated sAA and glycated sAA by fructose or MGO after 7 days. Each lane was loaded with de following sample: (a): sAA; (b): sAA+F; (c): sAA+MGO.
(TIF)

S9 Fig Enzymatic activity of non-glycated sAA and glycated sAA by fructose or MGO after 7 days. ** p < 0,01; *** p < 0,001.
(TIF)

S1 Raw images The original image of blot. (PDF)
