# Supplementary figures and images for: Fructose and methylglyoxal-induced glycation alters structural and functional properties of salivary proteins, albumin and lysozyme
Source: PLoS One. 2022 Jan 21;17(1):e0262369. doi: 10.1371/journal.pone.0262369 (PMC8782344; doi:10.1371/journal.pone.0262369)

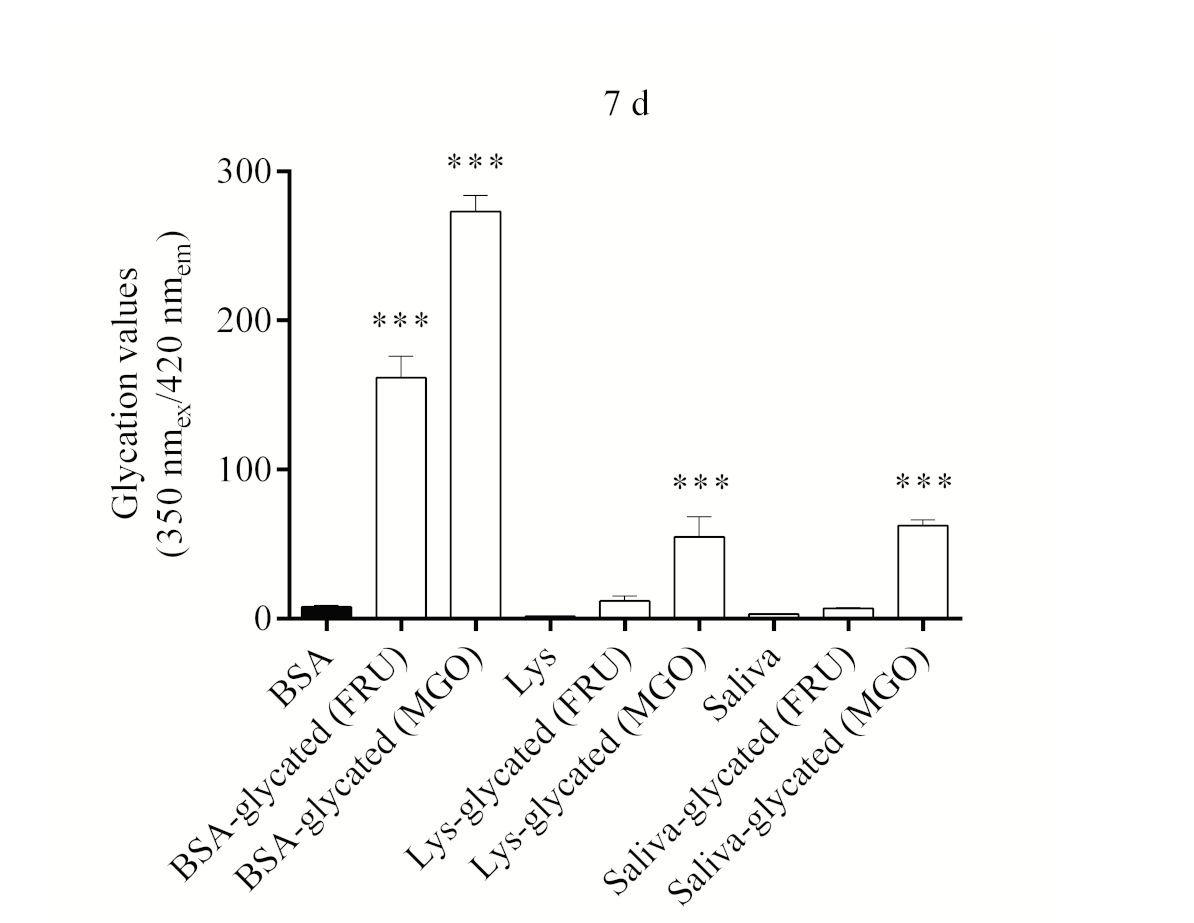

Supplement: S1 Fig — ** p < 0,01; *** p < 0,001. (TIF) [file pone.0262369.s001.tif]

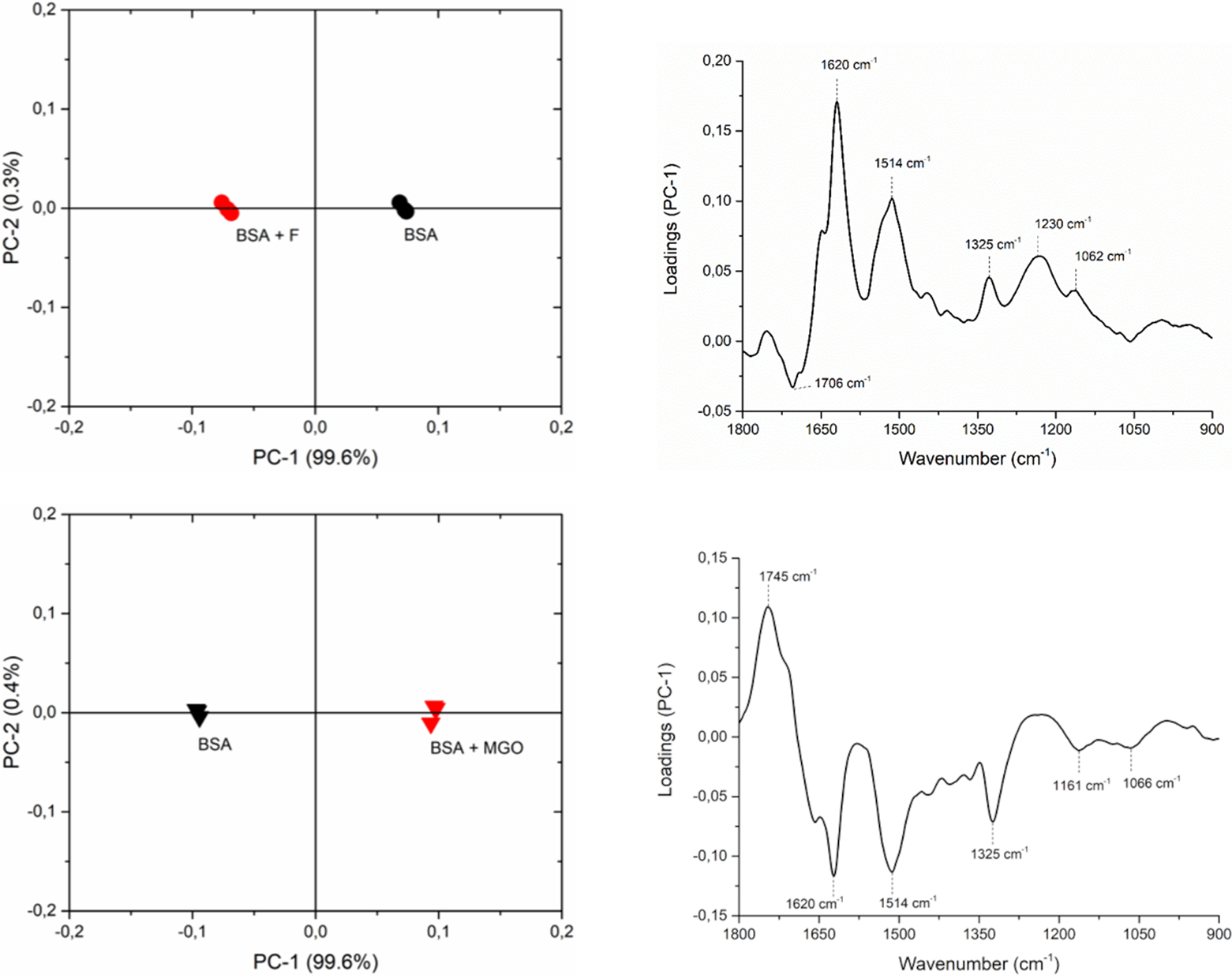

Supplement: S2 Fig — (A) Scores scatter plot of PC1 vs PC2 performed on the BSA and BSA+F spectrum. (B) PC1 loadings profile obtained for BSA and BSA+F. (C) Scores scatter plot of PC1 vs PC2 performed on the BSA and BSA+MGO spectrum. (D) PC1 loadings profile obtained for BSA and BSA+MGO. (TIF) [file pone.0262369.s002.tif]

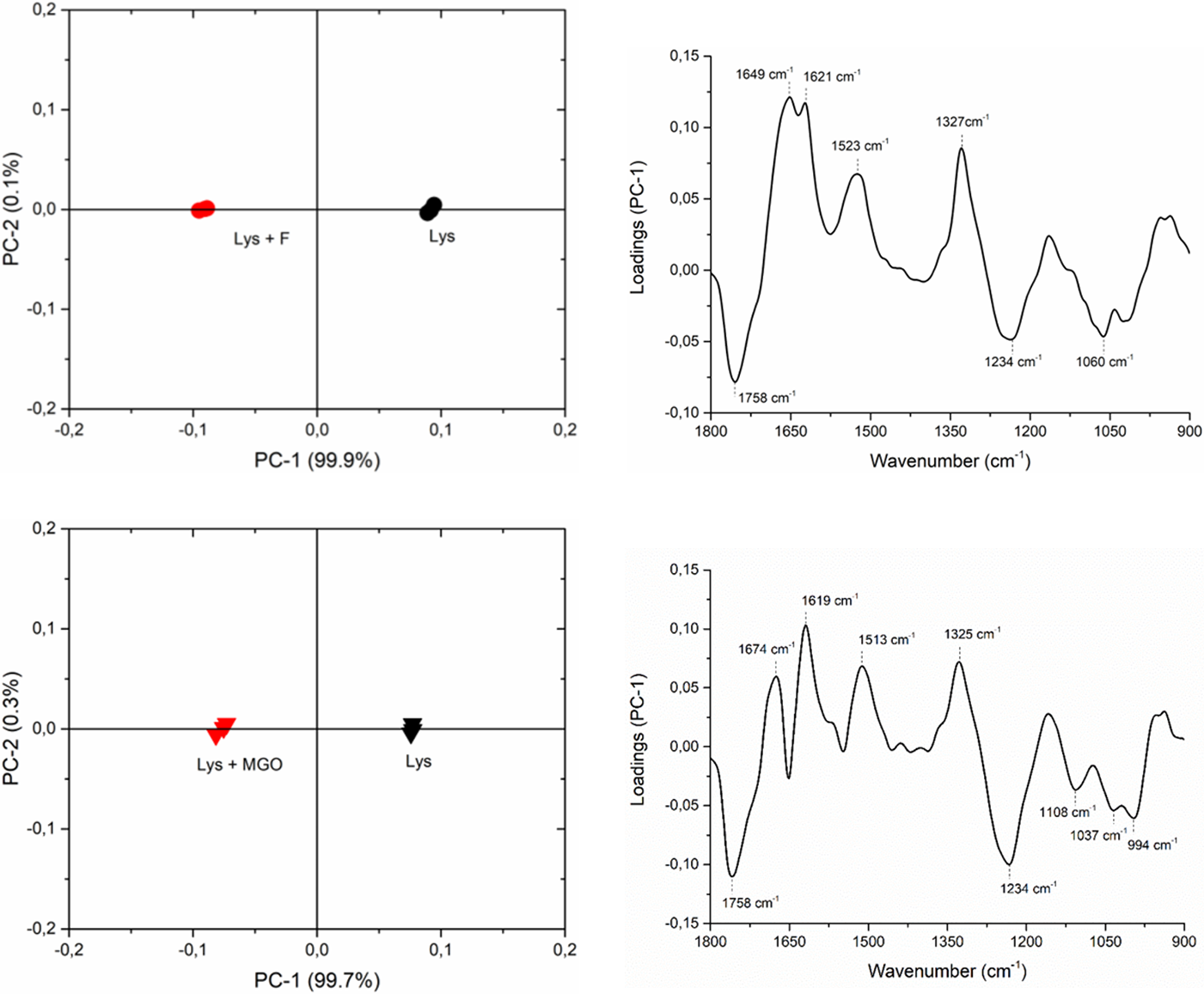

Supplement: S3 Fig — (A) Scores scatter plot of PC1 vs PC2 performed on the LYS and LYS+F spectrum. (B) PC1 loadings profile obtained for LYS and LYS+F. (C) Scores scatter plot of PC1 vs PC2 performed on the LYS and LYS+MGO spectrum. (D) PC1 loadings profile obtained for LYS and LYS+MGO. (TIF) [file pone.0262369.s003.tif]

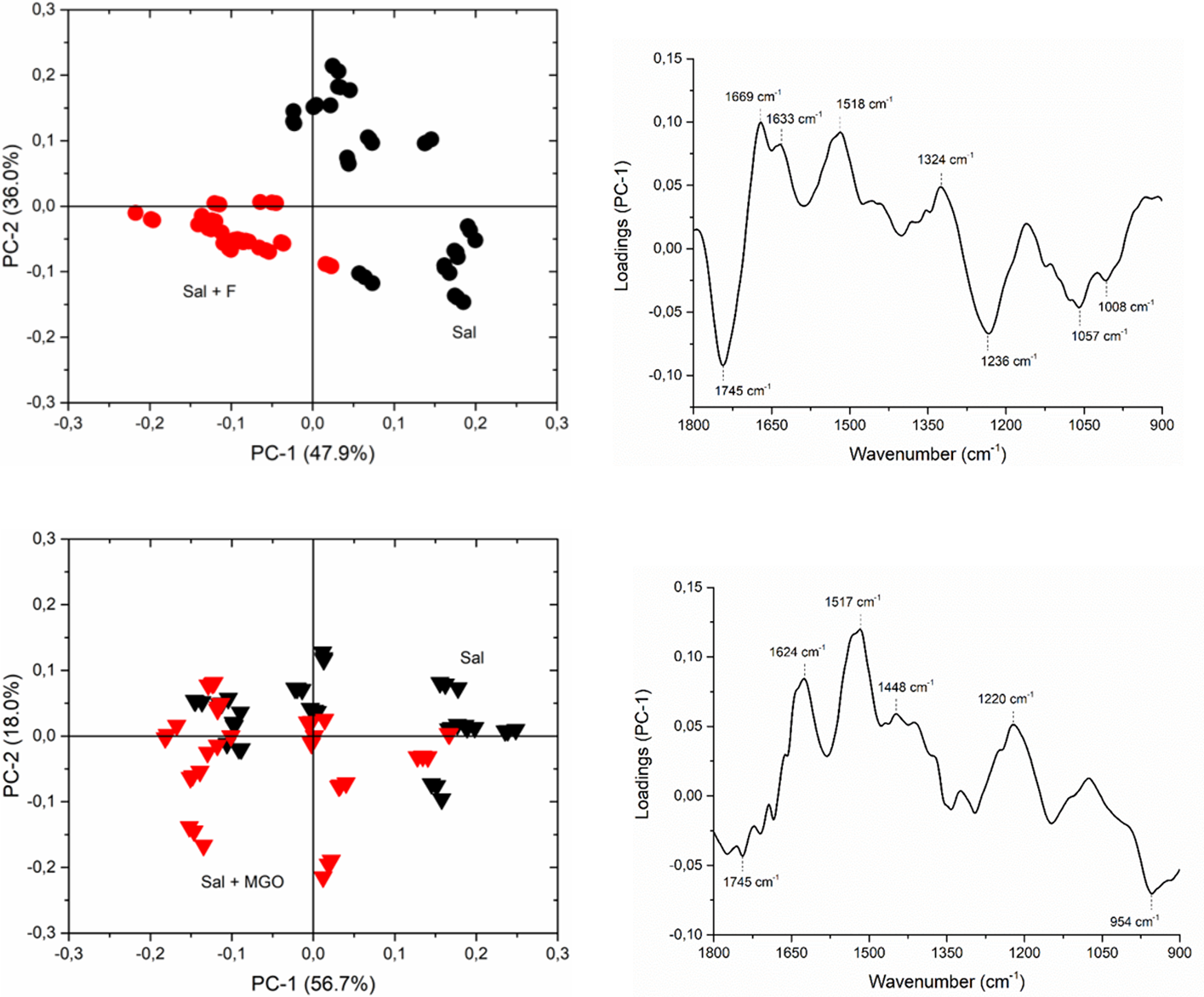

Supplement: S4 Fig — (A) Scores scatter plot of PC1 vs PC2 performed on the SAL and SAL+F spectrum. (B) PC1 loadings profile obtained for SAL and SAL+F. (C) Scores scatter plot of PC1 vs PC2 performed on the SAL and SAL+MGO spectrum. (D) PC1 loadings profile obtained for SAL and SAL+MGO. (TIF) [file pone.0262369.s004.tif]

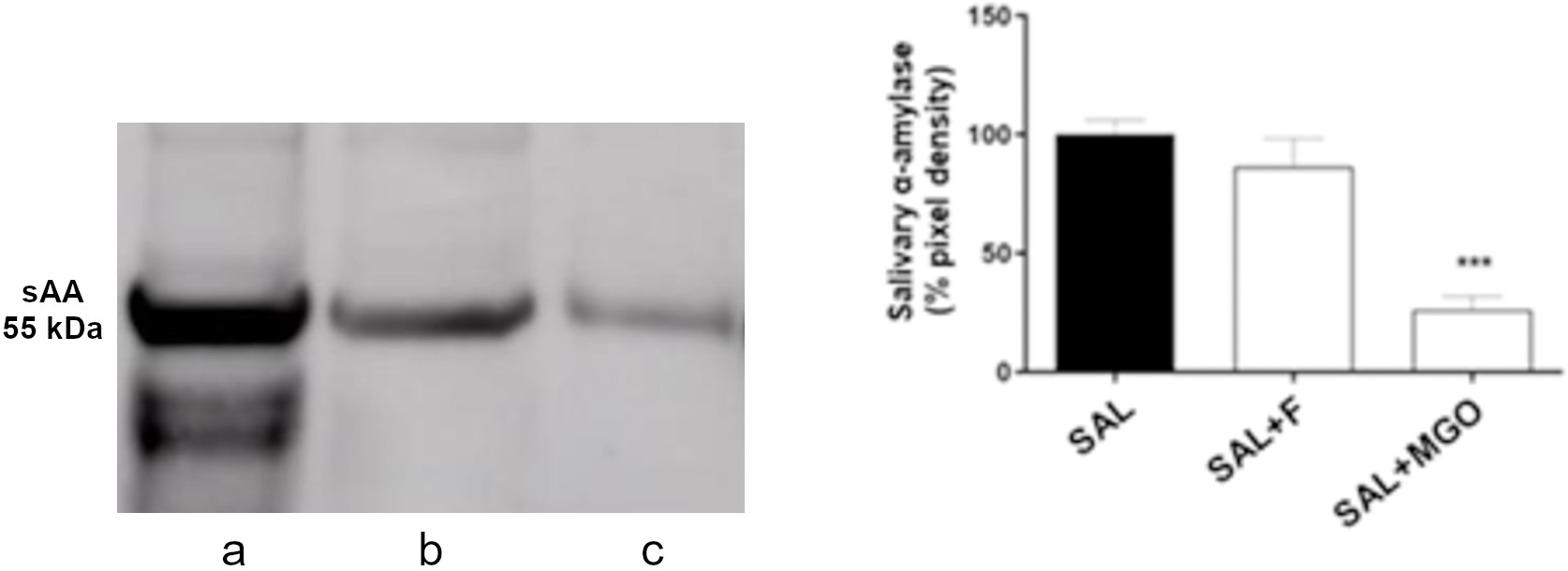

Supplement: S5 Fig — (A) Western blotting of sAA expression in SAL (lane a), SAL+F (lane b), and SAL+MGO (lane c). (B) Quantification of immunodetected sAA given in density of pixels. (TIF) [file pone.0262369.s005.tif]

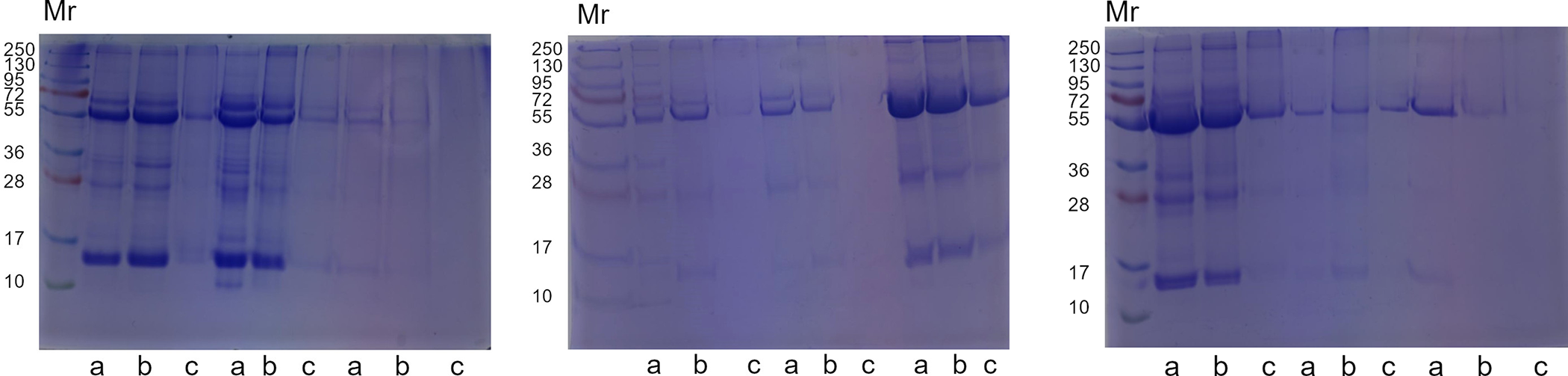

Supplement: S6 Fig — (A,B and C) Each lane was loaded with de following sample: (a): SAL; (b): SAL+F; (c): SAL+MGO; Mr: relative molecular mass of the protein standard (kDa). (TIF) [file pone.0262369.s006.tif]

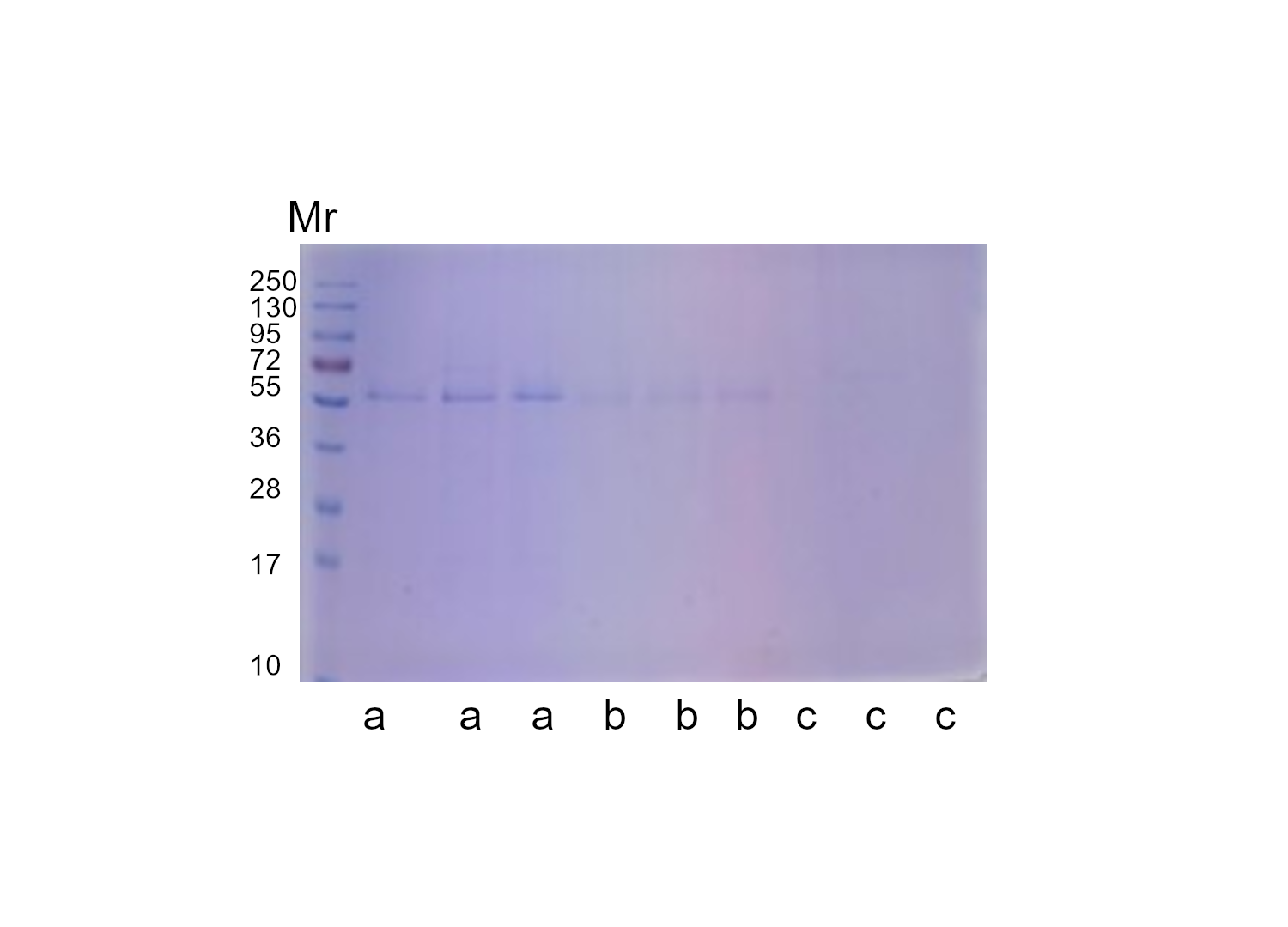

Supplement: S7 Fig — Each lane was loaded with de following sample: (a): sAA (b): sAA+F; (c): sAA +MGO. (TIF) [file pone.0262369.s007.tif]

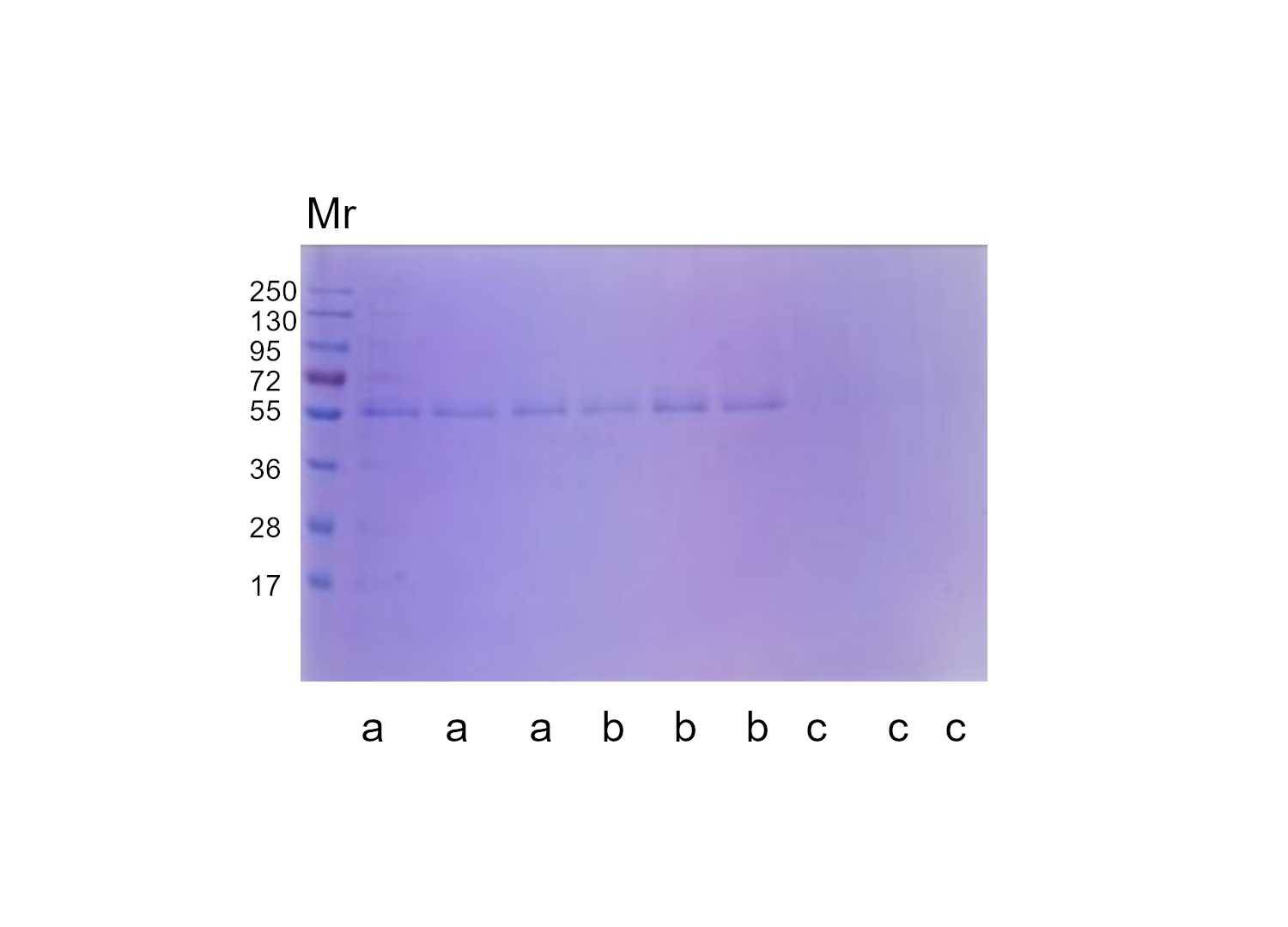

Supplement: S8 Fig — Each lane was loaded with de following sample: (a): sAA; (b): sAA+F; (c): sAA+MGO. (TIF) [file pone.0262369.s008.tif]

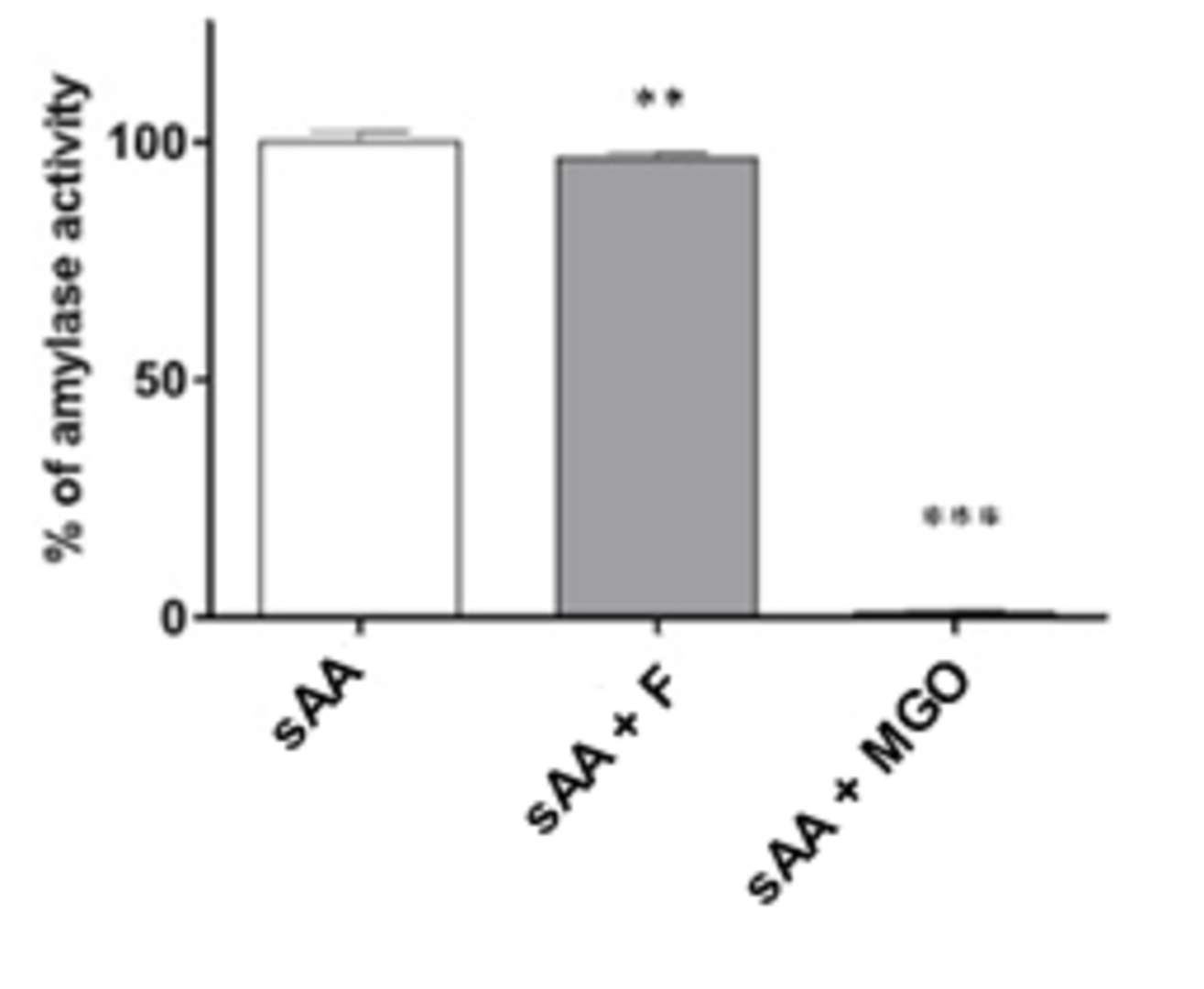

Supplement: S9 Fig — ** p < 0,01; *** p < 0,001. (TIF) [file pone.0262369.s009.tif]

The row image of S5A Fig.

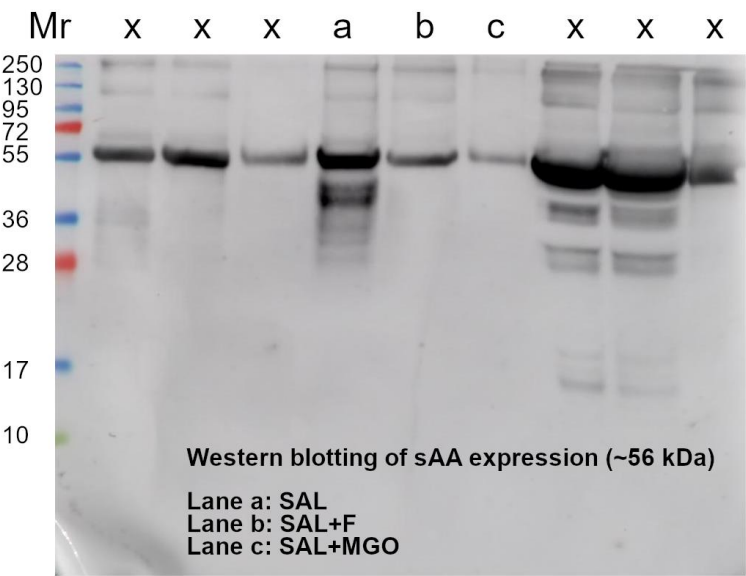

Supplement: S1 Raw images — (PDF) [file pone.0262369.s010.pdf]
